# Cytotoxic activity of caffeic acid and gallic acid against MCF-7 human breast cancer cells: An *in silico* and *in vitro* study

**DOI:** 10.22038/AJP.2019.13475

**Published:** 2019

**Authors:** Hasan Rezaei-Seresht, Hamid Cheshomi, Farahnaz Falanji, Fatemeh Movahedi-Motlagh, Maryam Hashemian, Erfan Mireskandari

**Affiliations:** 1 *Traditional and Complementary Medicine Research Center, Sabzevar University of Medical Sciences, Sabzevar, Iran*; 2 *Cellular and Molecular Research Center, Faculty of Medicine, Sabzevar University of Medical Sciences, Sabzevar, Iran*; 3 *Department of Biology, Faculty of Science, Ferdowsi University of Mashhad, Mashhad, Iran*; 4 *Department of Medical Genetics, Tehran University of Medical Sciences, Tehran, Iran*; 5 *Department of Nutrition, Faculty of Medicine, Sabzevar University of Medical Sciences, Sabzevar, Iran*; 6 *Digestive Oncology Research Center, Digestive Diseases Research Institute, Tehran University of Medical Sciences, Tehran, Iran*; 7 *Department of Chemistry, School of Sciences, Hakim Sabzevari University, Sabzevar, Iran*

**Keywords:** Caffeic acid, Gallic acid, MCF-7, P53, P21, Mcl-1

## Abstract

**Objective:**

Phenolic compounds have been considered inhibitors of various cancers.

**Material and Methods:**

In this study, caffeic acid and gallic acid were appraised for their possible effects on apoptotic genes expression in a breast cancer cell line *in vitro*. We also evaluated ligand interaction and ligand binding with estrogen receptor alpha by molecular docking. To determine half maximal inhibitory concentration, MCF-7 cells were treated with different concentrations of caffeic acid and gallic acid by 3-(4, 5-dimethylthiazol-2-yl)-2, 5-diphenyltetrazolium bromide assay. Furthermore, morphological changes in cells and alterations in *P53*, *Mcl-1* and *P21* gene expression were studied by real-time RT-PCR. Also, protein network and different interactions between the desired genes were analyzed using GeneMANIA database.

**Results:**

Evaluation of cell survival by MTT assay revealed that the half-maximal inhibitory concentration values for caffeic acid and gallic acid against MCF-7 cells, were 159 and 18 µg/ml, respectively. These compounds were found to affect *P53*, *Mcl-1* and *P21* gene expression; this alteration in gene expression probably occurred along with the activation of intrinsic apoptotic signaling pathway.

**Conclusion:**

Via apoptosis induction, caffeic acid and gallic acid have induce toxic effects and morphological changes in breast cancer cells, suggesting their possible future application as antitumor agents.

## Introduction

Breast cancer is the most common cause of cancer mortality (Brewster et al., 2014), with more than one million annual new cases of breast cancer diagnosed around the world (Miller et al., 2016). Breast cancer incidence differs across geographic locations, and the pattern of cancer incidence among migrating populations has been shown to resemble the host country. This suggests that there exist non-genetic, potentially modifiable lifestyle factors such as diet that may affect cancer development (Rosendahl et al., 2015).

Human and animal studies have revealed the roles of estrogen receptor (ER) in female and male sexual development and behavior, reproductive function, regulation of the neuroendocrine and cardiovascular systems and bone metabolism (Korach et al., 2003). The loss of ERα gene expression is a key factor in breast cancer progression, and it is associated with a more aggressive tumor phenotype and loss of sensitivity to endocrine therapy drugs such as tamoxifen (Singh et al., 2003), the most widely used anti-estrogen in clinical practice (Kurokawa and Arteaga, 2001; Zheng et al., 2015). However, tamoxifen is a commercially available drug, which binds the estrogen receptor and blocks its function and prevents the proliferation of breast cells.

Some of the most potent antiapoptotic factors influencing ERα expression and cancer cell survival, are the members of the *Bcl-2* gene family, including *myeloid cell leukemia-1*(*Mcl-1*). The oncogene *Mcl-1* is involved in hematogenous and solid tumor cell survival (Beroukhim et al., 2010). Down-regulation of *Mcl-1* is essential, but not sufficient, for apoptosis initiation (Pietrzak and Puzianowska-Kuznicka, 2008).

Many chemotherapeutic agents promote the expression of pro-apoptotic molecules such as P53 (Halazonetis et al., 2008), which regulates cellular stress via death receptor- and mitochondria-mediated apoptotic pathways (Vousden, 2000).

The expression of some molecular markers such as *P21 *in cancer cells determines their tumorigenic potential (Hanahan and Weinberg, 2000). *P21*^WAF1^^/^^CIP^ regulates cell cycle progression and acts as a general inhibitor of cyclin-dependent kinases (Yan et al., 2015). Lack of *P21* entails tumorigenesis along with other oncogenic mutations (Martín-Caballero et al., 2001; Poole et al., 2004; Yang et al., 2005). However, it was found that *P21* can negatively impact the activity of other positive regulators of the cell cycle, and the increasing expression of *P21* in tumor cells is a challenge to “braking” the procedure of cellular proliferation at the G1 checkpoint (Thor et al., 2000). The capacity of *P21* to promote cell cycle inhibition may depend on its capability to mediate *P53*-dependent gene suppression, as *P21* is both essential and sufficient for P53-dependent repression of genes regulating cell cycle progression (Löhr et al., 2003). Paradoxically, *P21* might promote apoptosis through both P53-dependent and P53-independent mechanisms under definite cellular stresses. The primary role of optimal anticancer agents is to kill tumor cells by triggering apoptosis signaling pathways (Tabuchi et al., 2009).

Phenolic acids such as caffeic acid (CA) (3, 4-dihydroxycinnamic acid) and gallic acid (GA) (3, 4, 5-trihydroxybenzoic acid) found in fruits, coffee and vegetables are potential anticancer, anti-inflammatory, antimicrobial, immunoregulatory, and antioxidant compounds (Rao et al., 2006; Olsen et al., 2009). Mechanisms suggested for their anticancer effects include stimulation of *P53* and *P21* gene expression and inhibition of CDK2 gene expression, which may lead to G0/G1 arrest in the cell cycle. Although recent studies have implicated these compounds in the development of a variety of cancers, the underlying mechanisms are yet to be elucidated (Skehan et al., 1990; Chang et al., 2002; Han et al., 2002; Ye et al., 2010; Nabavi et al., 2013). 

The objective of this study was to simultaneously study the anti-breast cancer effects of gallic acid and caffeic acid, as two important phenolic compounds, on the MCF-7 cell line. In addition, the effects of these compounds were investigated, for the first time, on *P53*,* P21 *and* Mcl-1 *gene expression in an ERα-positive breast cancer cell line *in silico* and *in vitro*, simultaneously. For the first time, we focused on the intrinsic apoptotic signaling pathway, as a major apoptosis pathway, and the relationship between these genes and tested compounds. Therefore, to explore mechanisms associated with CA- and GA-induced modulation of breast carcinogenesis, we assessed the ligand interaction and binding mode of these compounds with ERα using molecular docking, analyzed associated protein networks and gene interactions using GeneMANIA database, and investigated their effects on hormone receptor positive breast cancer cells (MCF-7), morphological alteration and proliferation by MTT (3-(4, 5-Dimethylthiazol-2-yl)-2, 5-diphenyltetrazolium bromide) assay. Ultimately, using real-time RT-PCR, we measured the effects of CA and GA on the expression of apoptotic genes, including *P53*, *Mcl-1* and *P21* in MCF-7 cell line.

## Materials and Methods

**Reagents**

CA and GA with the chemical structure shown in Figure 5A, dimethyl sulfoxide (DMSO), ethylene dinitrile tetra acetic acid (EDTA), 3-(4, 5-dimethylthiazol-2-yl)2, 5-diphenyltetrazoliumbromide (MTT), penicillin–streptomycin and trypsin were purchased from Sigma-Aldrich (USA). FBS and RPMI-1640 medium were purchased from Gibco (Scotland), and tamoxifen bought from Sigma-Aldrich (USA) and was used as a control drug. 

**Molecular docking**

The molecular docking of all compounds was done into the three-dimensional X-ray structure (PDB code: 3ERT). The AutoDock 4.2 software was used for the docking studies. This automated method is useful for studying the binding mode of ligands binding to biomacromolecules. The three-dimension structures of ligand molecules were built, optimized (PM3), and saved in PDB format using the molecular modeling program SPARTAN (Wavefunction Inc.). All non-protein atoms were deleted, and AutoDockTools was used for creating PDBQT files from primary PDB files. A grid box was constructed around the binding site of protein with number of grid points [npts]: X:60, Y:60, and Z:60 in the three dimensions, coordinates of three dimensions [gridcenter]: X:121.952, Y:90.314, and Z:5.066, and spacing of 0.375. Hydrogen atoms and Gasteiger partial charges were added using AutoDockTools (Ver. 4.2). In the present docking study, the Lamarckian genetic algorithm (LGA) method, implemented in the program AutoDock 4.2, was employed. All default docking parameters were maintained except for the maximum number of energy evaluations [ga_nim_evals] that was changed to 2.5×10^6^ and the number of runs [ga_run] altered to 100. AutoDockTools includes several methods for analyzing the results of docking simulations, including tools for clustering the results by conformational similarity, and visualizing conformations, interactions between ligands and proteins, and the affinity potentials created by AutoGrid. Results were visualized via PyMol (http://www.pymol.org).

**Preparation of compounds solutions**

To prepare different concentrations of CA and GA (5, 10, 30, 50, 75, 100, 130, 170, and 200 μg/ml), 2 mg of each compound was dissolved in 100 μl dimethyl sulfoxide and diluted with complete culture medium before the experiments. To better evaluate the various treatments, the viability of each group was compared with its relevant control containing equal amounts of DMSO.

**Culture of MCF-7 cells**

The human breast cancer cell line (MCF-7) was purchased from Pasteur Institute (Tehran, Iran). The cells were cultured in RPMI1640 medium (pH 7.2-7.4), supplemented with penicillin G (100 U/ml), streptomycin (100 U/ml) and 10% fetal bovine serum (FBS), and incubated at 37°C in a humidified atmosphere with 5% CO_2_. Cells were routinely sub-cultured using 0.25% trypsin and 1 mM EDTA. The human primers were commercially purchased form DENA ZIST Asia, Mashhad, Iran.

**Morphological alterations**

MCF-7 cells were treated with half maximal inhibitory concentration (IC_50_) dose of each compound and morphological changes were observed under a light inverted microscope (Olympus, Japan) following 48 hr of treatment.

**MTT assay**

The *in vitro* tetrazolium-based colourimetric assay (MTT) is a rapid method developed based on the cleavage of a yellow tetrazolium salt to purple formazan crystals by mitochondrial enzymes in metabolically active cells (Mosmann, 1983). The logarithmically growing MCF-7 cells were plated at a density of 10,000 cells/well into a 96-well plate. After 24 hr, cells were treated with CA at various concentrations (5, 10, 30, 50, 75, 100, 130, 170, and 200 μg/ml) for 48 and 72 hr. Also, the effect of tamoxifen citrate on the viability of cells was evaluated 48 and 72 hr after treatments. Next, 20 µl MTT (5 g/L) was added to each well and incubated for an additional 4 hr; then, culture media were then discarded and 150 μl DMSO was added. The absorbance was measured at 545 nm using an ELISA reader (Awareness, USA). The percentages of living cells were calculated against the controls according to the following equation for different treatments:

%viability of cells = the mean absorbance of treated cells in each well/the mean absorbance of control cells (DMSO) × 100. 

**Expression of** ***P53*** **,** ***Mcl-1*** **and** ***P21*** **in MCF-7 cell line assessed by Real-time PCR**

Total RNA was extracted using TRIzol reagent and used as a template for the production of cDNA which was employed in SYBR-based quantitative real-time PCR for the quantification of *P53*, *Mcl-1*, and *P21* transcript levels. QRT PCR reaction was carried out in thermocycler detection system (Bio-Rad, CFX96, USA) under the following conditions: 10 min at 95°C, 15 sec at 94°C, 30 sec at 55°C, and 30 sec at 72°C; each cycle was repeated 40 times. Primers used in this study are listed in Table 1. Using ∆∆CT method, we analyzed the real time PCR data, and all experiments were performed in triplicates.

**Table 1:** Primers used in this study

**Gene **	**Forward Primer**	**Reverse Primer**
***P53***	CCCCTCCTGGCCCCTGTCATCTTC	GCAGCGCCTCACAACCTCCGTCAT
***Mcl-1***	CCAAGAAAGCTGCATCGAACCAT	CAGCACATTCCTGATGCCACCT
***P21***	TGCCGAAGTCAGTTCCTTGTGG	CGCATGGGTTCTGACGGACATCC
***GAPDH***	ACCCAGAAGACTGTGGATGG	TCTAGACGGCAGGTCAGGTC

**Analysis of protein network using GeneMANIA database**

**Figure 1:**
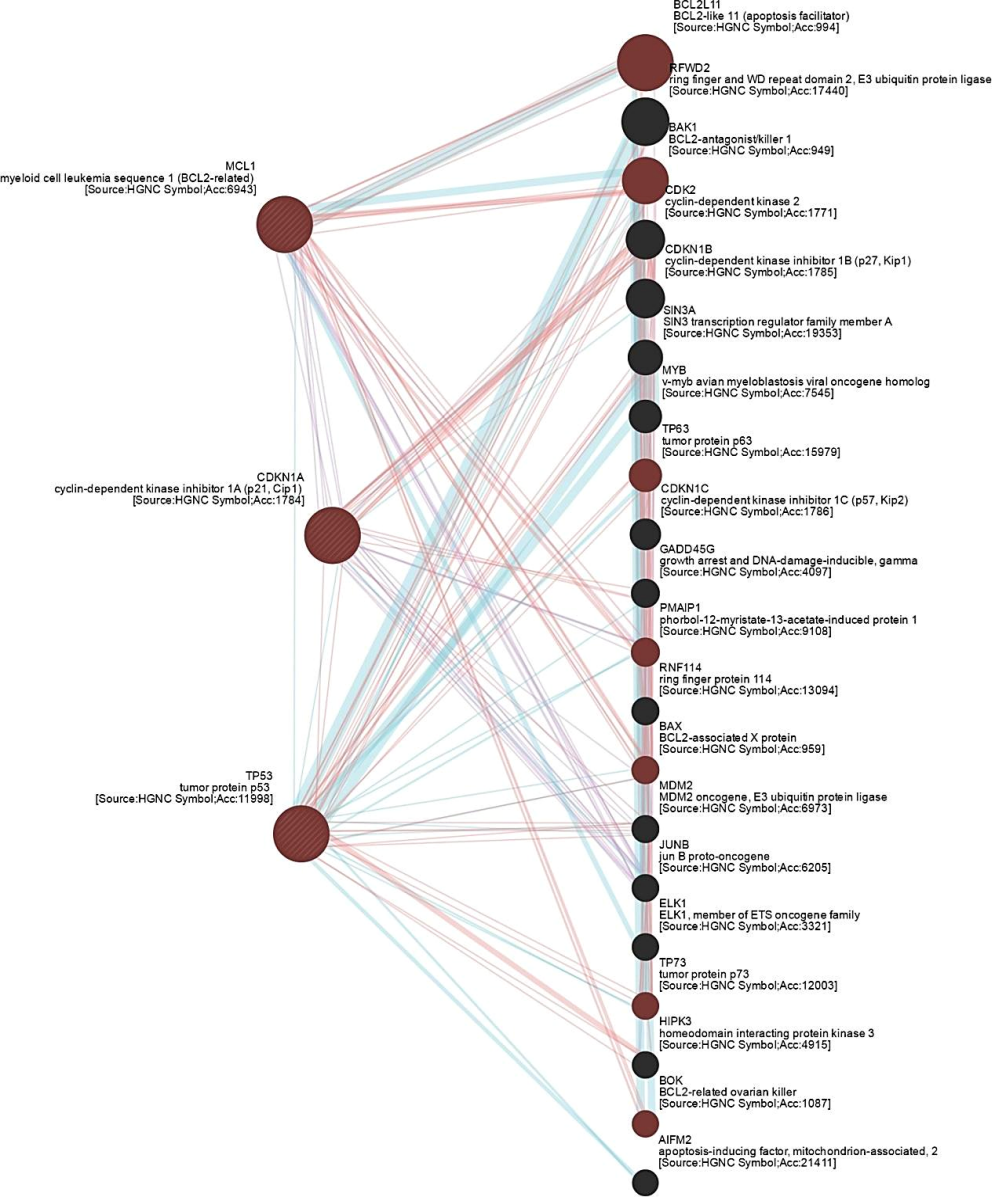
Network connectivity shows functional interaction between investigated genes and other genes associated with intrinsic apoptotic signaling pathway. *P53*, *Mcl-1* and *CDKN1* (*P21*) have a direct association regarding physical interactions, co-expression and signaling pathway. In each cluster network, display-colored lines indicate a direct link, blue lines indicate the link provided by the pathway, pink lines represent the physical interactions, and purple lines show interactions with co-expression. Pink circle labels the genes involved in intrinsic apoptotic signaling pathway

The GeneMANIA database, available at http://www.genemania.org/, calculates physical interactions, co-expression, predicted links, pathways, co-localization, genetic interactions, and shared protein domains at an expected level of accuracy. GeneMANIA database was employed to investigate the association between genes of interest and known interacting proteins in apoptotic signaling pathways, and understand the functional interactions. We analyzed all direct interactions networks between *P53*, *Mcl-1* and *CDKN1* (*P21*) genes (supplementary data). In each cluster network, display-colored lines indicate a direct link, blue lines indicate the link provided by the pathway, pink lines represent the physical interactions, and purple lines show interactions with co-expression (Figure 1).

**Statistical analysis**

The direct networks between *P53*, *Mcl-1* and *CDKN1* (*P21*) genes analyzed by GeneMANIA database. Furthermore, all data were analyzed using Kolmogorov–Smirnov test. Based on the normality test, data distribution in most experiments was normal. Differences were determined by one way ANOVA and Student's t-test (two-tailed), followed by Tukey multiple comparison test (normal data), and Mann–Whitney U test (non-normal data), using GraphPad software (Ver. 6). Values were expressed as mean±SD, and a p value less than 0.05 was considered statistically signiﬁcant.

## Results

**Molecular Docking**

Table 2 summarizes ligand-protein docking scores for caffeic acid, gallic acid, and tamoxifen with molecules on ERα. Tamoxifen showed binding interactions with an active residue of target molecule Arginine 394 (Arg394). Docking score of tamoxifen with the ER_α_ was -11.21 kcal/mol. Other properties of reference drug are given in Table 2.

**Table 2:** Docking scores of tamoxifen, CA and GA into ER-α

**Compounds**	**s-score** ^a^ **(kcal/mol)**	**Ki** ^b^	**H-bond interaction**
**Tamoxifen**	-11.21	61 nM	Arg-394, Glu-353
**Caffeic acid**	-4.47	524 nM	Arg Arg-394, 2H-Glu353
**Gallic acid**	-4.2	933 nM	Arg-394, Glu-353, Leu-346

^a ^S-score: binding free energy.

^b ^Ki = e^ ΔG/RT^, R = 1.986 cal/mol.K, T = 298 K

**Figure 2:**
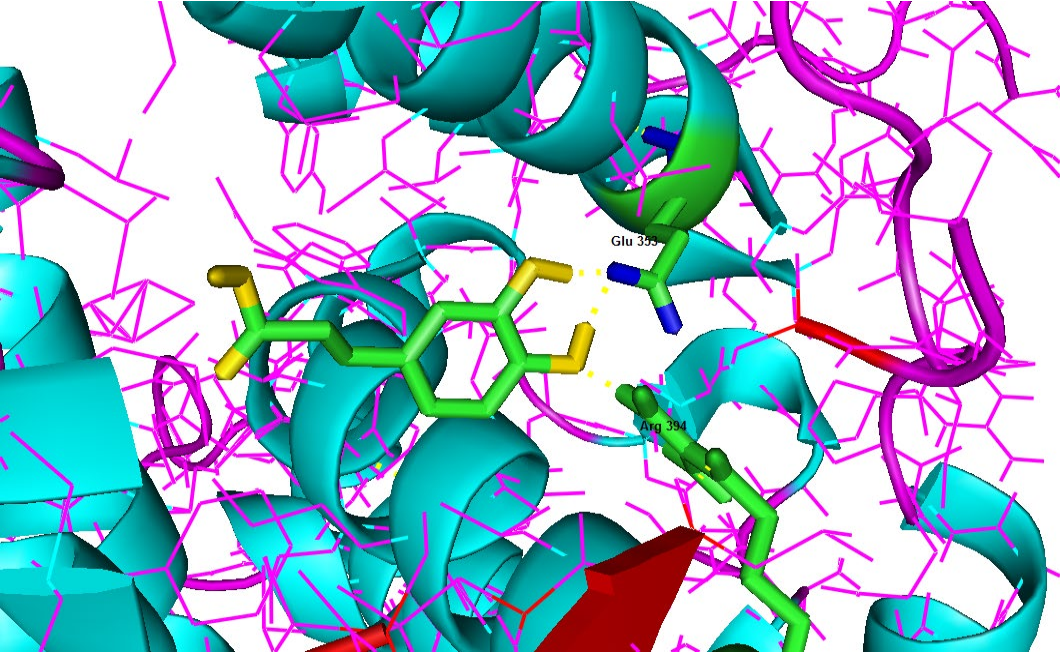
Ligand interaction and the binding mode of CA with human estrogen receptor alpha (entry 3ERT in the Protein Data Bank), exhibiting 2 H-bond with Glu 353 and 1 H-bond with Arg 394. The formed hydrogen bond is yellow

**Figure 3:**
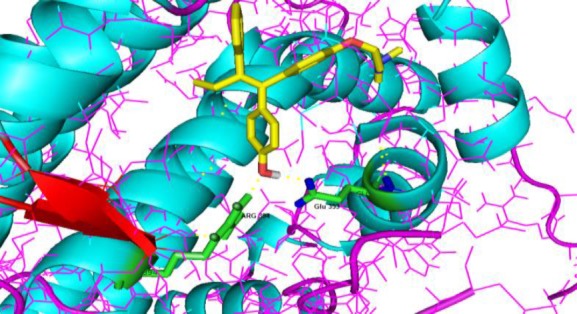
Ligand interaction and the binding mode of tamoxifen with human estrogen receptor alpha (entry 3ERT in the Protein Data Bank), showing 2 H-bond with Arg 394 and Glu 353. The formed hydrogen bond is yellow

**Figure 4:**
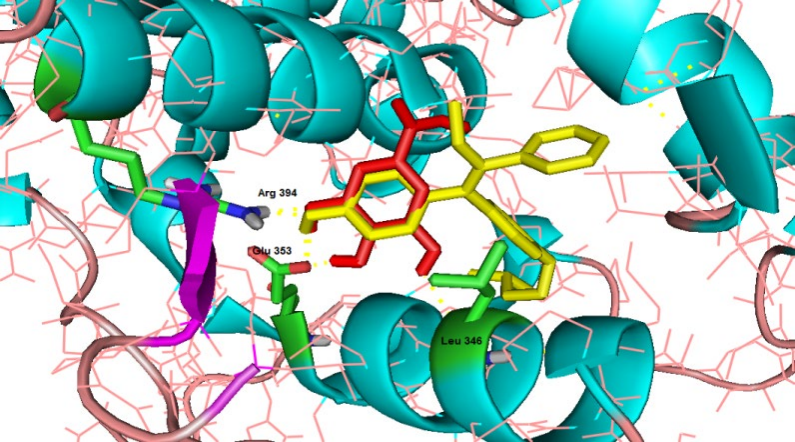
The binding mode of tamoxifen (yellow) and GA (red) with human estrogen receptor alpha (PDB code: 3ERT). Tamoxifen is connected to ERα via two H-bond (Arg 394 and Glu 353) and GA is connected to ERα via four H-bond with Arg 394, Leu 346, Glu 353. The hydrogen bonds are shown in yellow

Interactions and binding mode of caffeic acid, gallic acid, and tamoxifen with ERα, are illustrated in Figures 2-4. The docking results for caffeic acid and gallic acid (-4.47 kcal/mol and -4.2 kcal/mol, respectively) showed that these compounds reveals different mechanisms compared to the tamoxifen. Caffeic acid contains two hydroxyl groups in the ring that can bind ERα. Hydroxyl group of the *para* position of the ring binds Arg-394 and Glu-353, like tamoxifen, blocks ER functions, and prevents the proliferation of breast cells.

**Cytotoxic activity**

**Figure 5:**
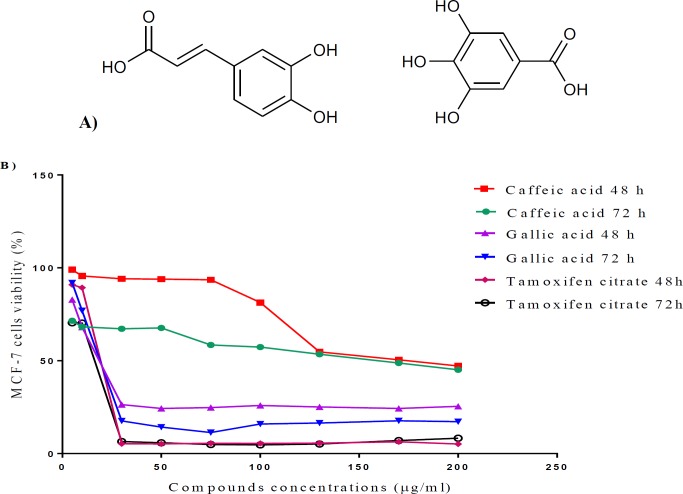
The chemical structures of CA and GA (left and right, respectively) (A); Time-based dose–response curves for MCF-7 during 48 and 72 hr treatments with CA and tamoxifen citrate (B)

To investigate anticancer activity, MCF-7 cells were treated with compounds at concentrations ranging from 5 to 200 µg/ml for 48 and 72 hr, and their viability was evaluated by MTT assay. CA and GA induced a time- and dose-dependent reduction in cell viability (Figure 5B). The IC_50_ values of compounds against MCF-7 cells were further determined (Table 3). To compare the cytotoxic effects of these compounds with an agent used against breast cancer, MCF-7 cells were also treated with a variety of tamoxifen citrate concentrations for the same periods. The IC_50_ values of tamoxifen citrate against MCF-7 cells were 19.4 and 16 µg/ml following 48 and 72 hr of treatments, respectively (Figure 5B). Our results indicated that CA and GA inhibited MCF-7 breast cancer cell proliferation in a dose-dependent manner and affected apoptotic gene expression. 

**Table 3:** IC_50_ values for CA, GA and tamoxifen citrate

**Compound**	**IC**_50_ ** (48 hr)**	**IC**_50_ ** (72 hr)**
**Caffeic acid**	170 µg/ml	159 µg/ml
**Gallic Acid**	18.5 µg/ml	18 µg/ml
**Tamoxifen citrate**	19.4 µg/ml	16 µg/ml

**Morphological alterations**

**Figure 6:**
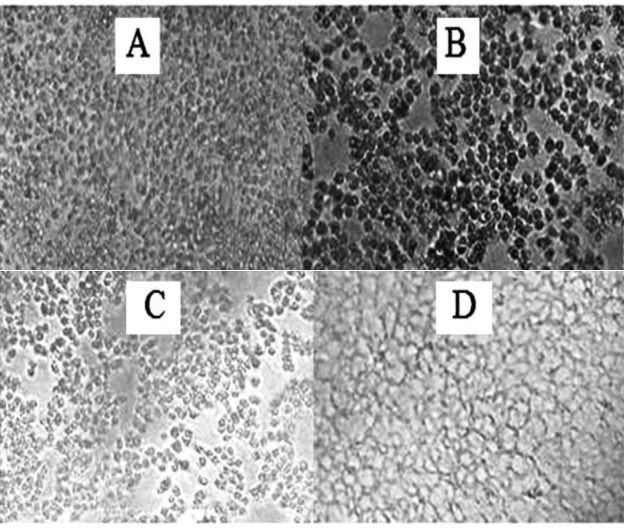
The morphological alterations of MCF-7 cells in non-treated cells (control) (A); after 48-hr treatment with 18 µg/ml of GA (B); 170 µg/ml of CA (C); and tamoxifen citrate

Morphological studies showed that the number of MCF-7 cells and eminent cytoplasmic granulations decreased following 48 hr treatment with CA and GA, in comparison with the untreated and control cultures. Notably, treatment with experimental compounds changed the morphology of MCF-7 cells similar to tamoxifen citrate (Figure 6).

**Gene expression**

**Figure 7:**
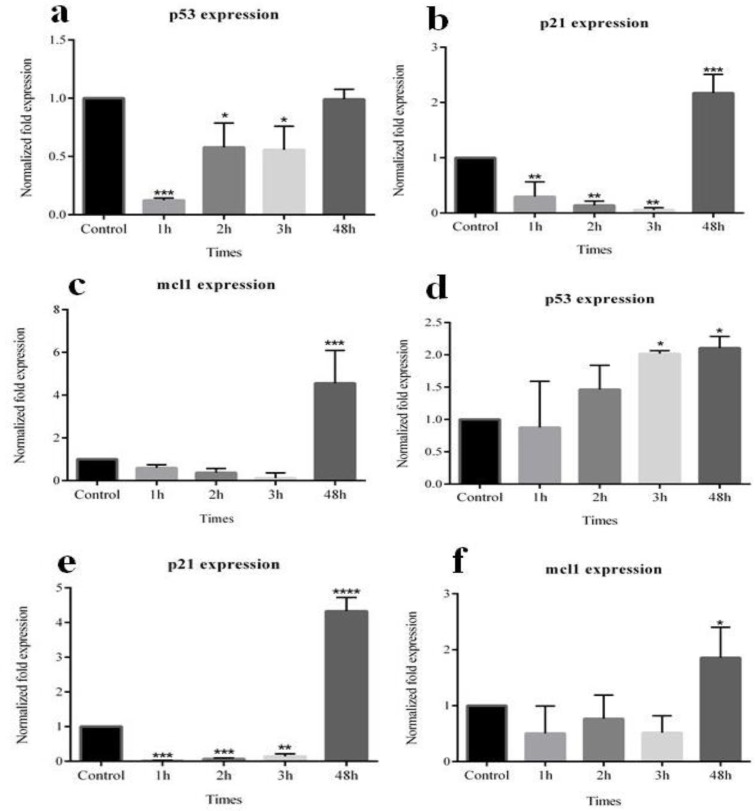
The effects of CA and GA on gene expression pattern as assessed by real-time RT-PCR. Upon CA treatment, *P53* (a), *P21* (b), and *Mcl-1* (c) and after GA treatment, *P53* (d), *P21* (e), *Mcl-1* (f) of MCF-7 cells were significantly affected. Asterisk indicates significant difference in mRNA expression in comparison with untreated cells used as the control group. *p<0.05, **p<0.01, ***p<0.001, ****p<0.0001

The possible roles of CA and GA in altering the expression of *P53*, *P21*, and *Mcl-1* genes in MCF-7 cells were further investigated. Results showed that CA had significant effects on the desired genes expression in the MCF-7 cells (including *P53* down-regulation in 1, 2, 3 hr, *P21* down-regulation in 1, 2, 3 hr, and up-regulation in 48 hr, and *Mcl-1* up-regulation in 48 hr). Our data showed that GA also significantly impacted the expression of *P53*, *P21*, and *Mcl-1* in MCF-7 cells (including *P53* up-regulation in 3 and 48 hr, *P21* down-regulation in 1, 2, 3 hr and up-regulation in 48 hr, and *Mcl-1*up-regulation in 48 hr). Figure 7 shows the changes in the expressions of *P53*, *P21* and *Mcl-1* genes.

**Interactions**

CA and GA targeted genes with a functional connection with intended genes involved in intrinsic apoptotic signaling pathway (*P53*, *Mcl-1*, and *P21*). The network also shows a correlation between intended compound-targeted genes and other genes effective in intrinsic apoptotic signaling pathway, such as *BCL2l11*, *BAK1*, *TP73*, *BOK*, *BAX*, *TP63*, and *PMAIP1*. By this analysis, we showed probable interactions between certain CA and GA regulated genes. 

## Discussion

Generally, the results of this study are in agreement with previously published researches, which also showed the anticancer effects of these compounds against different types of cancers including breast (Rosendahl et al., 2015), prostate (Sanderson et al., 2013), lung (Lin et al., 2012), colon (Chiang et al., 2014), and gastric cancer (Chang et al., 2002), as well as hepatocarcinoma (Guo et al., 2015) and fibrosarcoma (Prasad et al., 2011) for CA; other studies exhibited the anticancer effects of GA on colorectal cancer (Forester et al., 2014), hepatocellular carcinoma (Lima et al., 2016), and human oral cancer (Guimaraes et al., 2016).

To further clarify the possible mechanism of action of these compounds in the present research, we focused on the expression of a set of master genes associated with apoptosis in breast cancer. Network connectivity, for the first time, showed the functional interactions between investigated CA/GA-regulated genes and other genes associated with intrinsic apoptotic signaling pathway. It was also found that *P53*, *Mcl-1* and *P21* had a direct association concerning physical interactions, co-expression and signaling pathway; hence, we focused on the effect of CA and GA on the gene expression of *P53*, *P21* and *Mcl-1* in MCF-7 cell line. As specified in the results, after treatment with CA and GA, the expression of these genes was altered, reinforcing our hypothesis regarding the possible effects of these compounds on the intrinsic apoptotic signaling pathway.

*P53*, also known as *TP53*, codes a protein that functions as a tumor suppressor and partakes in the initiation of apoptosis process (Kerr et al., 1972; Huang and Strasser, 2000). In response to different forms of cellular stress, mitochondrial level of P53 increases (Erster et al., 2004). Based on the data from this study, it seems that CA does not incremental affect on *P53* gene, yet GA increases its expression. This gene induces apoptosis by target gene activation and transactivation-independent in mitochondria (Moll and Zaika, 2001). It has been recently found that GA induces apoptotic cell death, but not necrosis, in HepG2 cell line (Lima et al., 2016). Protein P53 mediates the DNA damage-related checkpoint through the transactivation of several growth inhibitory or apoptotic genes. Among these genes, the small 165 amino acid protein P21, also known as P21^WAF1/Cip1^, mediates P53-dependent G1 growth arrest (Abbas and Dutta, 2009). How *P21* helps apoptosis is not clear, but might depend on both P53-independent and p53-dependent upregulation of pro-apoptotic protein BAX, activation of the members of the tumor necrosis factor family of death receptors, or via affecting DNA repair (Gartel, 2005). Results of *P21* gene expression following cell treatment with CA and GA, showed that the overexpression of this gene occurred at different times post treatment. Growth arrest by *P21* can promote cellular differentiation, thereby inhibiting cell proliferation. Several other studies have shown that GA improves the viability of HL-60 cell line via *P21* overexpression, and suppression of apoptosis induced by the overexpression of caspase-3, caspase-8 and caspase-9 (Yeh et al., 2011). 

Furthermore, the overexpression of P21, by inhibiting several CDKs activity, such as CDK2, CDK3, and CDK4, ensues cell cycle arrest in G1 or G2 (Gartel and Tyner, 2002). P21 can also bind proliferating cell nuclear antigen (PCNA), thereby directly preventing DNA synthesis (Chen et al., 1995). Also, the expression of this key protein could be controlled by P53-dependent and -independent mechanisms at transcriptional, post-transcriptional, or post-translational levels (Zhu et al., 2004). However, DNA damages can lead to the activation of ATM/ATR-ERK pathway, which ultimately results in the induction of P21 expression in a P53-independent manner (Kim et al., 2002). Therefore, according to the data obtained from our study, it can be verified that in MCF7 treated with the desired compounds, P53 is unnecessary for P21 over-expression.

Moreover, Bcl-2 and its related proteins are among the most significant regulators of apoptosis (Williams and Cook, 2015). Various studies have established that *Mcl-1* mRNA is up-regulated as part of an early rapid cellular response to cytotoxic stimuli such as calcium ionophores, chemotherapeutic agents, pneumococcal infection and UV irradiation (Kafi et al., 2018). After studying the expression of this gene, we recognized that *Mcl-1*, unlike the two other genes, is slightly overexpressed following treatment with the two phenolic compounds; However, it is noted that MCL-1 is a major cause of resistance to chemo and radiotherapies, hence, inhibiting the expression of this gene is an important issue in cancer-related research. (Beekman and Howell, 2016) .

Furthermore, sub-apoptotic concentrations of cytotoxic compounds can induce growth arrest with senescence features, in which, *P53* and P*21*^WAF^^-1/CIP-1 ^are essential players; apoptosis plays an important role in myriad physiologic procedures in cell development; therefore, failure in apoptosis facilitates tumor formation and development (Jia et al., 2014; Seresht et al., 2019). Accordingly, it is possible to say that CA and GA are able to have different effects on the expression of such genes and the induction of apoptosis in treated cancer cells.

Findings of this study showed that MCF-7 cells treated with the intended compounds underwent several changes associated with cell death, such as morphological changes, and their numbers were reduced. Moreover, it was found that these compounds had significant effects on the expression of apoptosis genes such as *P53*, *P21* and *Mcl-1.* Accordingly, we suggest that these phenolic compounds are probable candidates for more research in animal models of breast cancer, as therapeutic agents, and adjuvants to standard chemotherapeutic drugs. By further cellular, molecular and *in vivo* studies on the effects and probable side effects of CA and GA, maybe in the not too distant future, these materials can be applied for possible health benefits as food supplements and medication for patients with breast cancer.
